# Lung Fibrosis after COVID-19: Treatment Prospects

**DOI:** 10.3390/ph14080807

**Published:** 2021-08-17

**Authors:** Evgeny Bazdyrev, Polina Rusina, Maria Panova, Fedor Novikov, Ivan Grishagin, Vladimir Nebolsin

**Affiliations:** 1 Research Institute for Complex Issues of Cardiovascular Diseases, 6, Sosnoviy Blvd., 650002 Kemerovo, Russia; 2 PHARMENTERPRISES LLC, Skolkovo Innovation Center, Bolshoi Blvd., 42(1), 143026 Moscow, Russia; p.rusina@pharmenterprises.ru (P.R.); m.panova@pharmenterprises.ru (M.P.); fnovikov@pharmenterprises.ru (F.N.); nv@pharmenterprises.ru (V.N.); 3 Zelinsky Institute of Organic Chemistry, Russian Academy of Sciences, 47 Leninsky Avenue, 119991 Moscow, Russia; 4 Rancho BioSciences, 16955 Via Del Campo Suite 200, San Diego, CA 92127, USA; grishagin@gmail.com

**Keywords:** COVID-19, pulmonary fibrosis, rehabilitation, nintedanib, pirfenidone, treamid, deupirfenidone

## Abstract

At the end of 2019, a highly contagious infection began its ominous conquest of the world. It was soon discovered that the disease was caused by a novel coronavirus designated as SARS-CoV-2, and the disease was thus abbreviated to COVID-19 (COVID). The global medical community has directed its efforts not only to find effective therapies against the deadly pathogen but also to combat the concomitant complications. Two of the most common respiratory manifestations of COVID are a significant reduction in the diffusing capacity of the lungs (DLCO) and the associated pulmonary interstitial damage. One year after moderate COVID, the incidence rate of impaired DLCO and persistent lung damage still exceeds 30%, and one-third of the patients have severe DLCO impairment and fibrotic lung damage. The persistent respiratory complications may cause substantial population morbidity, long-term disability, and even death due to the lung fibrosis progression. The incidence of COVID-induced pulmonary fibrosis caused by COVID can be estimated based on a 15-year observational study of lung pathology after SARS. Most SARS patients with fibrotic lung damage recovered within the first year and then remained healthy; however, in 20% of the cases, significant fibrosis progression was found in 5–10 years. Based on these data, the incidence rate of post-COVID lung fibrosis can be estimated at 2–6% after moderate illness. What is worse, there are reasons to believe that fibrosis may become one of the major long-term complications of COVID, even in asymptomatic individuals. Currently, despite the best efforts of the global medical community, there are no treatments for COVID-induced pulmonary fibrosis. In this review, we analyze the latest data from ongoing clinical trials aimed at treating post-COVID lung fibrosis and analyze the rationale for the current drug candidates. We discuss the use of antifibrotic therapy for idiopathic pulmonary fibrosis, the IN01 vaccine, glucocorticosteroids as well as the stromal vascular fraction for the treatment and rehabilitation of patients with COVID-associated pulmonary damage.

## 1. Introduction

*The single biggest threat to man’s continued dominance on the planet is the virus.*

                           Joshua Lederberg

The outbreak of a novel coronavirus strain, SARS-CoV-2, in December 2019 triggered the ongoing pandemic of the acute respiratory coronavirus-induced disease 2019 (COVID-19, also simply referred to as COVID) [1]. As the new treatments and approaches towards the reduction of disease mortality emerged, new data hinted at a “hidden pandemic” of long-term sequelae of severe COVID [2]. Observational studies indicate that 90% of COVID patients experience COVID sequelae such as respiratory problems, decreased exercise tolerance, and lung tissue damage [3]. These conditions are resolved within 3 months of hospitalization in 50% of the patients [3,4]. However, between 3 and 6 months after hospitalization, the rate of full recovery drops to 35%, and between 6 and 9 months, it goes down to just 15% [5]. The rate of complete recovery from lung damage specifically is 50% during the first 6 months after hospitalization and 75% during a 9-month period. At 9 months following hospitalization, 30% of the patients experience at least some COVID sequelae [5]. Furthermore, the majority of that population has residual lung tissue damage, with nearly a third (or 10% of all patients) exhibiting pronounced fibrotic lung damage; the majority of the patients in the latter group experience severe respiratory pathology and decreased exercise tolerance. Further observation did not reveal any trends in the reduction of the severity or frequency of COVID sequelae [5].

Elderly people with severe coronavirus symptoms or dangerous pre-existing health conditions are at the highest risk for post-COVID fibrosis (although the term “fibrosis” applies to irreversible disease, there is no consensus about the type of “fibrotic-like” sequalae in post-COVID patients [6]. Hence, in literature post-COVID fibrosis can be described as either organizing pneumonia [7], or interstitial lung disease [8,9], or just pulmonary fibrosis [10] or fibrotic lung disease [11] in general. Thus, here, by the term “post-COVID fibrosis”, we mean any “fibrotic-like” conditions.) [12]. Fibrosis was clinically confirmed in 56% of the patients who experienced moderate COVID symptoms and in 71% of the patients with severe symptoms, 3 months after they recovered from COVID [13]. Similar results were presented by Francone: fibrotic traces visible on a CT scan were found in 40.8% (53 out of 130) early disease phase and in 53.6% (70 out of 130) at a later stage [14]. According to Vasarmidi [15] and Rai [16], the rate of COVID-induced fibrosis may exceed 30%.

Despite a higher chance of fibrosis in intensive care patients, fibrosis has been also documented in patients who did not need artificial lung ventilation [17,18]. Thus, post-COVID de novo interstitial lung disease was found in a case study of five patients 92who were either asymptomatic or had very mild symptoms. These patients had symptoms of dyspnea, intermittent cough, and lingering fatigue 4–8 weeks after COVID diagnosis, consistent with the lung disease patterns observed on CT scans [19].

Currently, there is an active discussion about the possibility of spontaneous resolution and the need for drug treatment of persistent fibrotic lung injuries in post-COVID patients. Studies carried out on a number of respiratory infections such as influenza and atypical pneumonia confirm a clear link between the viral damage to lung tissues, the development of an aberrant inflammatory response, the formation of permanent lesions, and the occurrence of fibrosis [20]. In particular, the presence of persistent COVID-induced fibrotic lung damage one year after COVID is associated with a severe respiratory pathology and concomitant symptoms [5], and at an earlier stage (3–5 months after COVID), with inflammatory response complications in the respiratory tract tissue [21,22,23]. It is plausible that the prolonged inflammatory response causes further damage to the vascular endothelium and epithelium of the respiratory tract and leads to cytokine-induced tissue damage. In turn, this may prevent the restoration of normal respiration and lung tissue regeneration [21]. The upregulation of TGF-β1 and other growth factors (FGF, EGF), followed by the activation of pro-fibrotic pathways and the renin–angiotensin system imbalance, may also contribute to the development of post-COVID lung fibrosis [24].

The dynamics of the long-lasting sequelae in patients who have recovered from severe COVID indicate that there is a 30% chance of developing a persistent respiratory system pathology and a 10% chance of developing a severe pathology. This includes the severe disruption of respiration, reduction of exercise tolerance, and the concomitant development of persistent fibrotic lung damage. Unfortunately, to date, there are no data that would allow one to reliably estimate the chances of the pathology progression and the development of COVID-induced lung fibrosis 10 years after COVID recovery. Therefore, it is reasonable to forecast the future rate of COVID-induced lung fibrosis using the data of the patients who have recovered from other coronavirus infections such as SARS. More importantly, SARS and COVID have similar pathogenetic features, analogous dynamics in the resolution of the sequelae during the first 12 months after diagnosis as well as comparable rates of respiratory pathologies (23.7% for SARS and 33% for COVID) and persistent fibrotic lung damage (27.8% for SARS and 38% for COVID) [25,26]. Based on the 15 years of observational data on SARS patients, it has been established that the severity and prevalence of the respiratory pathology and the residual lung damage are reduced within the first 12 months after recovery [25,27], but remain unchanged in the next 14 years [28]. Another set of observational data allows one to conclude that the risk of developing fibrosis after recovery from a SARS infection is ca. 20% [25,26,28,29,30]. Thus, for patients who have recovered from moderate to severe COVID, we can estimate the risk of developing fibrosis at 2–6%. In turn, this means that the estimate for the prevalence of COVID-induced fibrosis is somewhere between 10 and 15 patients per 10,000 people in the general population, which is 10–100 times higher than the risk of idiopathic lung fibrosis.

## 2. Clinical Trials of Therapies for Post-COVID Lung Fibrosis

As of January 2021, there are a total of 11 drugs, two technologies, and one vaccine undergoing clinical trials for the treatment and prevention of lung fibrosis in COVID survivors. In this review, we consider the agents approved by the U.S. Food and Drug Administration (FDA) such as nintedanib [31] or pirfenidone [32] for idiopathic pulmonary fibrosis (IPF) and other therapies. Table 1 compiles the information registered in the ClinicalTrials.gov [33] database by January 2021.

More detailed information on drugs in clinical trials for the treatment of lung fibrosis associated with COVID is provided below.

### 2.1. Nintedanib and Pirfenidone

Nintedanib and pirfenidone are antifibrotic medicines that, despite different mechanisms of action, are equally efficacious in inhibiting the reduction of respiratory function by ~50% and increase the life expectancy of IPF (idiopathic pulmonary fibrosis) patients by 2.5 years on average [50]. Nintedanib (6-methoxycarbonyl-substituted indolinone) is an oral therapeutic used for the treatment of IPF and a second-line treatment for non-small cell lung adenocarcinoma. In 2020, this drug was approved for the treatment of advanced chronic fibrotic interstitial lung diseases.

Lung fibroblasts from IPF patients and in vivo models demonstrate that the antifibrotic activity of nintedanib is associated with the inhibition of pro-fibrotic mediators, including platelet-derived growth factor (PDGF) and fibroblast growth factor (FGF), transforming growth factor beta (TGF-β), and vascular endothelial growth factor (VEGF). Nintedanib binds to the intracellular ATP pockets of the corresponding receptors, which leads to the inhibition of pro-fibrotic signaling and the attenuation of the proliferation, migration, and differentiation of fibroblasts as well as extracellular matrix component secretion [51].

Pirfenidone (5-methyl-1-phenyl-2-(1H)-pyridone) is an oral antifibrotic agent with multiple effects such as the regulation of pro-fibrotic and pro-inflammatory cytokine cascades and the inhibition of fibroblast proliferation and synthesis of collagen, which is a standard therapy for lung cancer and moderate IPF [52]. Pirfenidone decelerates fibrosis by inhibiting pro-fibrotic and pro-inflammatory cytokine cascades, including TGF-β signaling, which plays a central role in IPF pathogenesis [53]. Pirfenidone blocks TGF-β-stimulated collagen production, inhibiting the activation of HSP47 and Col1 genes. In in vitro and in vivo studies, pirfenidone showed an anti-inflammatory effect by suppressing the production of tumor necrosis factor-α (TNF-α), interferon gamma (IFN-γ), interleukin-1beta (IL-1β), and interleukin-6 (IL-6). Pirfenidone was also shown to have antioxidant properties; depending on the concentration, it blocked NADPH-dependent microsomal lipid peroxidation in the liver. Based on in vivo models, it was determined that pirfenidone suppresses TGF-β-associated fibroblast differentiation in the lungs [54].

At Present, there is no evidence that nintedanib or pirfenidone affects the severity of COVID symptoms. Furthermore, the side effects of these drugs are similar, in part, to those of COVID (e.g., diarrhea, fatigue, loss of appetite), which can hamper early diagnosis and worsen clinical manifestations [55]. Acute complications are some of the most serious IPF consequences, and the rate of in-hospital lethality for this condition exceeds 50%. Such acute IPF complications may be caused by respiratory viral infections [56]. A fraction of patients with acute IPF complications had viral infection symptoms, including human coronavirus OC43 infection, which was established by viral nucleic acid testing [57]. Although both antifibrotic agents have pleiotropic effects, neither of them is immunosuppressive. Thus, there is no evidence supporting the suspension of their use during an ongoing viral infection [50].

As of April 2020, pirfenidone and nintedanib have been commercially sold only in an oral form, and therefore have limited use among patients undergoing artificial lung ventilation. However, in December 2020, pirfenidone was used in patients with COVID-induced severe ARDS; the compound was administered through a nasogastric tube [58]. In February 2020, a clinical trial was initiated to evaluate the safety and efficacy of pirfenidone in new coronavirus patients >18 years old. During the four-day trial, the authors assessed the dynamics of damaged lung areas using chest CT scans, oxygenation, changes in blood gas content, and quality of life according to the King’s Brief Interstitial Lung Disease (K-BILD) questionnaire. Moreover, the researchers analyzed the mortality rate, clinical manifestation dynamics (dyspnea and coughing), blood parameters such as lymphocyte counts, viral nucleic acid, and markers of inflammation in the blood [37].

Another clinical trial of pirfenidone in patients with fibrotic changes after COVID was launched in August 2020 [38]. The established inclusion criteria selected (1) adults older than 18, (2) who had verified SARS-CoV-2 infection (3) that led to severe pneumonia and ARDS (4) with convalescence and/or clinical and radiological signs of pulmonary fibrosis on a high-resolution CT (HRCT) scan (with fibrotic changes of no less than 5% after recovery). This trial aimed to study how pirfenidone affected COVID-induced fibrotic changes, the level of forced vital capacity (FVC) of the lung, if it lowered oxygen uptake during exercise, increased exercise tolerance during the 6-min walking test (6MWT), requests for hospitalization (general as well as associated with respiratory disease), requests for emergency or outpatient care due to respiratory diseases, lung transplants, and mortality.

The first clinical trial of nintedanib started in April 2020. A single-center, randomized, placebo-controlled trial on the efficacy and safety of nintedanib for the treatment of lung fibrosis in patients with moderate and severe COVID symptoms was initiated. The cohort included patients 18–70 years old suffering from fibrosis of both lungs after recovery from COVID. The primary efficacy endpoint was the FVC measurement after eight weeks of therapy; the secondary endpoints were DLCO levels, 6MWT parameters, and HRCT eight weeks after therapy [34].

Phase III trial of nintedanib began in October 2020 [35]. The study included patients 18–89 years old and verified to have had COVID (positive PCR or serologic test results within the previous 2–6 months), with radiological signs (CT) of fibrosis (>10% of lung capacity) and DLCO ≤ 70%. The primary objective is to assess the efficacy of nintedanib in the retardation of lung fibrosis progression in COVID survivors expressed as FVC levels in 12 months compared to placebo. The authors aim to compare the rate of DLCO decrease after 6 and 12 months, exercise tolerance after 12 months, the increase in fibrotic changes (HRCT) after 12 months, and health-related quality-of-life changes; to evaluate dyspnea dynamics, changes in depression and anxiety levels, biomarkers of lung damage, lung hypertension and inflammation, rate of lung hypertension after 12 months compared to the moment of inclusion; to assess the link between genetic predisposition (MUC5B polymorphism) and lung fibrosis in COVID survivors, and the safety of the compound.

The third clinical trial of nintedanib (phase IV; in progress since November 2020) is aimed at investigating the influence of nintedanib on slowing down lung fibrosis in patients over 18 years old, with infiltrates or progressive lung damage appearing on chest X-rays or CTs not less than 4 weeks after the emergence of the first symptoms, and an FVC ratio below 80% or DLCO less than 50% of normal values [36]. Primary endpoints include FVC change in 180 days compared to the initial value. Secondary endpoints are death at 90 and 180 days after trial inclusion due to respiratory causes; a visual evaluation of the chest by CT scan; changes according to St. George’s Respiratory Questionnaire (SGRQ), K-BILD, Leicester Cough Questionnaire (LCQ), and others.

Data indicate a higher risk of thromboembolia of the pulmonary artery in COVID patients. Anticoagulant therapy may improve outcomes in patients with severe COVID and coagulopathy [59]. This observation is relevant to individuals receiving nintedanib, as it is likely that this drug may increase the risk of bleeding when used together with anticoagulants. In this case, antifibrotic therapy can be suspended to minimize the side effects of pharmacotherapy [50]. Additionally, pirfenidone and nintedanib may contribute to hepatotoxicity, while patients infected with SARS-CoV-2 often experience liver dysfunction. Elevated levels of liver enzymes were observed in 168 out of 757 patients (22%) with confirmed COVID and in 56/142 (39%) patients with severe symptoms [60]. The simultaneous use of antibiotics can increase the likelihood of liver dysfunction, which may be mitigated by a temporary suspension of antifibrotic therapy for in-hospital IPF patients with severe COVID and abnormal hepatic function tests until the levels of key liver function indicators are normalized [50].

### 2.2. Treamid

Treamid is also known as bisamide derivative of dicarboxylic acid (BDDA). Treamid caused anti-inflammatory and antifibrotic effects and lung tissue regeneration in animals with experimental pulmonary fibrosis by suppressing the production and deposition of collagen and stimulating endothelial progenitor cell synthesis. Preclinical data suggest that Treamid efficiently suppresses inflammation and restores the diffusing capacity of the lungs. These results are promising for the application of this drug as an antifibrotic therapeutic and in the restoration of the structure and function of the lungs in patients suffering from IPF and novel COVID-induced fibrosis [61].

Clinical trials of treamid started in September 2020 and are aimed at the evaluation of the safety and rehabilitation efficacy of the medication in patients who recovered from COVID-induced pneumonia [39]. The primary objective of the study is to assess the efficacy of treamid in changing FVC and/or DLCO during week 4 from the start of the treatment. Secondary objectives include the evaluation of safety and pharmacokinetic parameters of the compound, spirometric indicators (forced expiratory volume during the first second, FEV1, FVC, FEV1/FVC), body plethysmography (total lung volume, thoracic volume), DLCO and 6MWT analysis, scale of dyspnea (according to Borg and mMRC scales), quality of life according to K-BILD, and fibrotic change dynamics based on lung CT scan data.

### 2.3. LYT-100 (Deupirfenidon)

LYT-100 (deupirfenidon) is an N-aryl-pyridinone derivative with an undisclosed formula [62]. It is a deuterium-substituted analogue of pirfenidone that demonstrates anti-inflammatory and antifibrotic activity. LYT-100 is designed to be a potential treatment for IPF, interstitial lung diseases with fibrosis, respiratory COVID complications and related consequences, as well as lymphatic Bessel disorders. In September 2020, a Phase II clinical trial of LYT-100 was initiated to estimate its safety and efficacy in patients >18 years old with pneumonia (with at least two lung sections affected) [40]. The primary endpoint of the study is the distance walked during the 6MWT test on day 91. Additionally, pharmacokinetics, inflammatory biomarkers, dyspnea score on the Borg scale, and SF-36 quality of life will be evaluated.

### 2.4. Collagen-Polyvinylpyrrolidone (FibroquelMR)

In August 2020, phases I and II of clinical trials were launched for the collagen-polyvinylpyrrolidone (collagen-PVP) solution for intramuscular injections as a drug candidate for suppressing the cytokine storm in COVID patients [41]. It had been determined that collagen-PVP decreases levels of IL-1β, IL-8, TNF-alpha, TGF-β1, IL-17, Cox-1, leukocyte adhesion molecule (ELAM-1, VCAM-1 and ICAM-1), lowers the expression of other inflammatory mediators, and increases the level of IL-10 and the amount of Treg cells. Moreover, the drug candidate stimulates the suppression of tissue fibrosis during rheumatoid arthritis and osteoarthritis, and is not associated with any adverse events [63,64]. The trial included patients >18 years old with COVID symptoms (coughing, mucus production, odynophagia, dyspnea with or without fever, and radiological evidence of inflammatory infiltrates); positive test for SARS-CoV-2 by RT-PCR was not required. Criteria for inclusion are: individuals with laboratory predictors of mild and severe COVID symptoms (D-dimer > 1000 ng/mL; total number of lymphocytes < 800 cells/μL, creatine phosphokinase more than two times above the upper limit of normal range; troponin and ferritin > 300 mcg/L), SpO2 < 92%, and negative intradermal collagen-PVP reaction [41]. Primary endpoints include the need for oxygen therapy, mitigation of symptoms, and the recovery of lymphocyte levels. Secondary endpoints are the decrease of serum IP-10, anti-inflammatory cytokines in blood serum (TNF-a, IL-1β, IL-7), circulating effector T cells, lowered parenchymal attenuation, appearance of ground-glass opacity, pulmonary nodules, interlobular septal thickening, and/or bronchial wall thickening.

### 2.5. Glucocorticosteroids (Prednisone)

It was discovered that corticosteroids can mitigate the short- and long-term effects of COVID-induced pneumonia [65], and the prolonged use of corticosteroids may be beneficial in reducing the risk or the severity of subsequent post-COVID pulmonary fibrosis [2]. It was shown that prednisone can slow down the progression of pulmonary fibrosis in rat IPF models, and its mechanism may be related to the elevation of caveolin-1 levels and the reduction of TNF-a, TGF-b1, and PDGF levels [66].

Thus, corticosteroids may improve the symptoms of post-COVID pulmonary fibrosis by decreasing inflammation in the lungs. Confirming this hypothesis, a case series of three post-COVID patients revealed that a 1-month course of prednisone treatment resulted in a mild clinical improvement (reduced oxygen consumption at home, improvement on chest X-ray scans) with no major adverse effects [67]. Moreover, steroids are the accepted first-line treatment of organizing pneumonia, which was shown to be the prevailing condition in post-COVID patients having interstitial lung disease, with significant functional deficit 6 weeks after discharge (4.8%, 35 out of 837 survivors) [68]. Thirty of these patients received prednisone, resulting in significant symptomatic and radiological improvement.

A randomized controlled trial evaluating the efficacy of a low dose (20 mg) of prednisone, used in conjunction with symptomatic therapy for 14 days to treat lung infiltrates after COVID, was started in September 2020 and has been completed [42]. The trial included patients recovered from COVID with persistent radiological (CT) evidence of infiltrates. Efficacy criteria were the resolution of pulmonary infiltrates on a CT scan.

### 2.6. Bovhyaluronidase Azoximer (Longidase)

Bovhyaluronidase azoximer is a long-acting hyaluronidase synthesized via the conjugation of the corresponding enzyme with the copolymer of N-oxide 1,4-ethylene piperazine and (N-carboxymethyl)-1,4-ethylene piperazinium bromide. Antifibrotic properties of bovhyaluronidase manifest in the disruption of collagen fiber formation through the degradation of glycosaminoglycans by hyaluronidase. A clinical trial in 45 patients suffering from cryptogenic fibrosing alveolitis with concurrent pneumofibrosis demonstrated the benefits of bovhyaluronidase azoximer, namely, substantial alleviation of clinical symptoms (coughing and fatigue), and a 10% decrease in the spread and intensity of characteristic CT scan ground-glass opacity. Baseline therapy included oral glucocorticosteroids with an average dose of 15–20 mg daily; 25 (56%) patients also received the cytostatic drug azathioprine [69].

A clinical trial of bovhyaluronidase azoximer efficacy and safety for the prevention and treatment of post-inflammatory fibrosis and interstitial lung disease after COVID started in June 2020 [43]. The objective of the study is to compare the outcomes related to post-inflammatory fibrosis and interstitial lung disease in adults with post-COVID pulmonary complications in the group receiving bovhyaluronidase azoximer for treatment or prevention, and in the group of patients under dynamic observation. This trial includes patients aged over 18 years, with residual pulmonary changes due to COVID complications, discovered no later than 2 months from disease onset [43]. Primary trial efficacy endpoints involve the assessment of the fibrotic lung tissue damage and interstitial changes evidenced by the HRCT data at 2.5 months compared to the initial values. Secondary endpoints include lung fibrosis and interstitial changes, ground-glass opacity, hydrothorax, consolidation of images analyzed by Botkin.AI software (artificial intelligence); FVC and DLCO changes, rate of dyspnea according to mMRC scale, SpO_2_, 6MWT data, SpO_2_ dynamics after 6MWT; changes in residual and total lung capacity [43].

### 2.7. BIO 300 (Genistein)

BIO 300 is a patented synthetic genistein nanosuspension. Genistein (5, 7-dihydroxy-3-(4-hydroxyphenyl)-4h-chromen-4-one) is a soy isoflavone with very low bioavailability [70]. The structure of genistein is similar to that of estrogen; therefore, genistein is known as a phytoestrogen. Genistein binds all three estrogen receptors but has selective affinity to estrogen receptor beta (ER-β). There are several reports on the genistein-induced activation of cell cycle inhibitors p21 and p27 as well as GADD (the growth arrest and DNA damage) genes. Genistein also suppresses the expression of transcription factors (NF-kB) and c-Myc, and can contribute to cell cycle arrest by activating ER-β with the subsequent inactivation of NF-kB or by directly inducing the expression of cell cycle inhibitor proteins.

BIO 300 was initially developed to resist high radiation exposure and was eventually licensed by Humanetics Corporation for clinical oncology as a radiomodulator [70]. BIO 300, when administered after radiation exposure, lowered the number of micronuclei in the bone marrow and primary lung fibroblasts, which indicates the reduction of radiation-induced DNA damage [70]. Preclinical testing in mice aimed at evaluating the capacity of BIO 300 to reduce radiation-induced pneumonitis/fibrosis demonstrated a notable rise in survival to over 180 days (26.6%) as compared to untreated irradiated controls [71]. In addition to the enhanced survival, BIO 300 mitigated radiation damage and improved the overall lung function. Furthermore, genistein reduced collagen deposition by 28% in mice lungs taken at 4 week intervals, post-irradiation [72].

Notably, the characteristics of radiation-induced pneumonitis are similar to COVID-induced interstitial pneumonia [73,74]. For example, an increase in local neutrophils, cytokines, and other immune factors is seen both in patients and animals after acute radiation exposure and in COVID patients with lung damage [75,76]. Pneumonitis, which often progresses to pulmonary fibrosis [77], and a subsequent drop in blood oxygen levels are also observed both after irradiation and in COVID patients [60]. Therefore, treatments for radiation exposure could conceivably prove to be efficacious in treating post-COVID complications [2].

As genistein could inactivate NF-kB [78], which has been shown to reduce inflammation in a COVID mouse model, it could be effective in treating lung complications caused by the SARS-CoV-2 infection.

Since November 2020, BIO 300 has been tested for the treatment of pulmonary fibrosis in patients over 18 years of age and previously hospitalized due to COVID [44]. The primary study endpoint is a change in DLCO and 6MWT indicators after 12 weeks of treatment. Secondary endpoints include the change in spirometry parameters (FVC, FEV1, FEV1/FVC), SpO_2_, assessment of lung fibrosis signs on a chest CT scan on the 4-point Likert scale, SGRQ dynamics, monitoring of a range of biochemical blood test parameters, and mortality. Tertiary endpoints are changes in additional oxygen consumption duration, nightly additional oxygen consumption at rest and during exercise, TGF-β1 expression, and cytokines IL-1b, IL-6, IL-8, and TNF-α.

### 2.8. Tetrandrine

Tetrandrine (TET) is a major active component of the Chinese *Stephania tetrandra* S. Moore plant. TET has been described as a natural alkaloid with multidirectional action, affecting reactive oxygen species, calcium channels, and caspase-dependent pathways [79]. Due to its antitumor [79], anti-inflammatory [80], and antiviral activity against the Ebola virus (in vivo) and human coronavirus OC43 (in vitro) [81], TET has been tried as a treatment for rheumatism, lung cancer, and silicosis. TET can lower type I and III collagen mRNA and collagen deposition in lungs [80]. Recent data indicate that the agent can suppress lung fibrosis by autophagy activation, partially due to the transmission of Rheb/mTOR/p70S6K signals, as demonstrated in fibroblasts of mice with bleomycin-induced (BLM) lung fibrosis [82].

TET and a tetrandrine-hydroxypropyl-β-cyclodextrin (TET-HP-β-CD) obtained by tetrandrine lyophilization are efficacious against lung fibrosis in in vivo models through inhalation. TET and TET-HP-β-CD alleviated inflammation and fibrosis (verified by histological examination), limited the accumulation of hydroxyproline in the lungs (by 28% and 41%, respectively), and improved post-surgery survival (by 28% and 42% compared to the fibrosis control group, respectively) [83]. The appropriate aerodynamic diameter of drug particles should be approximately 1–5 μm for optimal administration to deep lungs [84], and both prepared formulations met this requirement. Inhaled TET and TET-HP-β-CD provided consistently higher pulmonary concentrations for 3 h after administration compared to TET administered intravenously [83]. It is therefore expected that a standard treatment regimen in combination with TET would decrease the rate of pulmonary fibrosis during post-COVID rehabilitation. Phase IV of the TET clinical trials started in March 2020 and included patients 18–75 years old with mild and severe pneumonia symptoms [45]. The primary endpoint is mortality.

### 2.9. Fuzheng Huayu Tablet (FZHY)

Fuzheng Huayu (FZHY, traditional Chinese medicine) is a blend of six herbs [85]. The formulation has been approved for the treatment of liver fibrosis by the China Food and Drug Administration [86]. FZHY is efficacious in suppressing the activity of matrix metalloproteinase 2, shown to participate in IPF development in an in vivo model [87] as well as suppressing type IV collagen expression in the lung [88]. These effects may be explained by one of the constituents of FZHY, hederagenin (HDG) [89]. HDG reduces BLM-induced lung dysfunction in a dose-dependent way. In addition, HDG decreases BLM-associated collagen deposition by lowering the levels of alpha-smooth muscle actin, type I collagen, inflammatory cytokines TNF-α and IL6, TGF-β1, and connective tissue growth factor [90]. Further analysis of the mechanism showed the ability of HDG to block Ras/JNK/NFAT4 signal transduction, which controls type I collagen expression in mesenchymal cells during lung fibrosis formation [91]. These mechanisms set the stage for a trial proposed to assess the efficacy of FZHY against post-COVID fibrosis in combination with pulmonary rehabilitation and vitamin C intake [46]. A double-blind, placebo-controlled, randomized, multicenter trial of FZHY started in April 2020 and included 18–65-year-old patients with pulmonary fibrosis after standard COVID treatment. Primary endpoints include the assessment of pulmonary function (FVC, DLCO), 6MWT, and changes in HRCT data. Secondary efficacy endpoints are dynamics of SpO_2_, experienced discomfort, quality of life, and SARS-CoV-2-specific IgG and IgM levels [46].

### 2.10. Anluohuaxian

Anluohuaxian is recommended for the treatment of liver fibrosis in China. Anluohuaxian is known to suppress the expression of type I and III collagen mRNAs, the tissue inhibitor of matrix metalloprotease-1 (TIMP-1), and TGF-β1 in patients with liver fibrosis [92]. Additionally, the pathogeneses of liver fibrosis and lung fibrosis are known to be quite similar [93]. Therefore, the efficacy and safety of anluohuaxian in the rehabilitation of patients with post-COVID lung fibrosis has been assessed. A multicenter, open, randomized, controlled trial started in April 2020 [47] and included patients with confirmed COVID and HRCT-supported pulmonary fibrosis diagnosis. The primary clinical efficacy endpoints are the changes in radiological manifestations determined using HRCT and in exercise tolerance measured by 6MWT. Secondary endpoints include the change in the physiological index, parameter dynamics according to SGRQ, dyspnea score on the mMRC scale, and lung vital capacity.

### 2.11. Stromal Vascular Fraction (cSVF)

Regenerative medicine based on stromal vascular fraction cells (cSVF) derived from adipose tissue is a new promising therapeutic for the treatment of IPF [48]. Heterogeneous SVF cell population consists of innate immune system cells (neutrophils, macrophages, monocytes, dendritic cells, natural killer cells, mast cells, etc.), which are essential for an adaptive immune system. According to data acquired by Alexander R. W., introducing autological cSVF can mitigate the inflammatory damage via the activation of the adaptive immune system in the damaged lung tissue without serious adverse effects [94]. Thus, the use of cSVF—known to help mitigate damage from severe inflammatory disorders, provide immunomodulation, and promote repair and regenerative effects—could have beneficial effects in fibrotic lung disorders [95].

Ntolios et al. demonstrated that the endotracheal administration of stromal cells can lead to considerable functional reduction of fibrosis in 24 months after the first injection [96], with a 6.2% change in DLCO (*p* = 0.04, 18 months) and a 6% change in FVC (*p* = 0.029, 24 months). Median total survival without progression was 26 months, and median total survival was 32 months. All patients survived for at least 2 years after the first injection. Twelve patients (85.7%) died of disease progression. Despite the encouraging data presented above, the results were obtained in a short-term, non-randomized trial without a placebo control [96].

A case study by Michalek et al. of a 71-year-old female patient suffering from IPF demonstrated that the intravenous administration of autological cSVF can contribute to lung regeneration without any serious adverse events [97]. Nine months after the therapy, the patient displayed a considerable improvement of the lung function (FVC 61–104%, FEV1 59–92%), with the positive effect lasting for 14 months post-treatment (FVC 102%, FEV1 97%).

The GARM-COVID trial initiated in March 2020 presupposes the intravenous injection of cSVF to improve irreversibly damaged alveolar tissue. The trial includes patients who recovered from COVID, with lung tissue damage confirmed by HRCT. The primary efficacy endpoint for cSVF use is the rate of adverse event incidence; the secondary endpoint is the analysis of radiological dynamics in the lung (HRCT), and exercise tolerance measured by 6MWT [48].

### 2.12. IN01 Vaccine

The IN01 vaccine is a hybrid of the recombinant epidermal growth factor (EGF) and cholera toxin B-subunit domain G33D (CTB-G33D). It produces the immune response in the form of polyclonal anti-EGF neutralizing antibodies [49]. It is assumed that the vaccine inhibits the binding of EGF to its receptor, which is overexpressed in IPF patients [98]. EGFR signaling mediates the hyperactive host response to lung injury during SARS-CoV-2 infection, which may contribute to the development of fibrosis [99]. Supposedly, the IN01 vaccine may be able to prevent lung fibrosis after SARS-COV-2 infection by blocking EGFR activation as well. Therefore, a COVINVAK trial was initiated in June 2020 [49]. The trial included patients >18 years old, who suffered from pneumonia, pulmonary dysfunction, and fibrotic changes associated with SARS-CoV-2 infection (according to the chest CT), and had previously undergone a non-invasive lung ventilation and had EGF levels of less than 200 pg/mL. The primary efficacy endpoint is safety (frequency and severity of adverse events). Secondary efficacy endpoints include blood oxygen level, quality of life assessed by the QoL questionnaire, and fibrotic change dynamics measured by HRCT.

### 2.13. Chitotriosidase Inhibitor OATD-01

In March 2020, OncoArendi Therapeutics announced the clinical trial of the chitotriosidase inhibitor OATD-01 as a treatment for post-COVID pulmonary fibrosis [100]. Chitotriosidase (chitinase 1) was shown to enhance TGF-β1-stimulated fibrotic responses [101]. However, the level of chitotriosidase expression in the lung tissue of patients with COVID is still under discussion [102]. Phase I of OATD-01 clinical trials are currently close to completion and thus far, the drug candidate has been demonstrated to be safe in healthy volunteers. The trials are conducted in Germany, and we were unable to locate them in the ClinicalTrials.gov database.

## 3. Conclusions

The novel coronavirus-induced disease led to a pandemic that poses a global threat to human health. The monitoring of patients who have recovered from COVID-associated pneumonia demonstrates that the significant reduction in DLCO and associated fibrotic signs in the lung parenchyma are factors associated with a negative prognosis. The persistent respiratory complications may cause substantial population morbidity, long-term disability, and even death due to lung fibrosis progression. The incidence rate of post-COVID lung fibrosis can be estimated at 2–6% after moderate illness. According to this estimation, the prevalence of post-COVID lung fibrosis will be from 10 to 30 patients per 10,000 populations, which is 30 times higher than the IPF prevalence.

The medical community is currently facing a lack of efficacious treatment options for COVID-induced fibrosis and is therefore compelled to look at the repurposing and repositioning of drugs and drug candidates. We believe that anti-inflammatory treatment for 6 months after COVID-associated pneumonia will reduce residual lung inflammation and improve the impaired diffusing capacity of the lungs. In turn, this will boost lung tissue regeneration and prevent persistent respiratory pathology. We hope that the clinical trials presented above will result in the discovery of efficacious tools to combat pulmonary fibrosis, one of the major COVID complications.

## Figures and Tables

**Table 1:** Clinical trials of drugs for the treatment of post-COVID lung fibrosis.

Treatment	NCT Number		Phase	Number Enrolled	Study Design
Nintedanib	NCT04338802	[34]	II	96	Single-center, randomized, placebo-controlled 150 mg POBID for 8 weeks
	NCT04541680	[35]	III	250	Single-center, randomized, placebo-controlled 150 mg POBID for 12 months
	NCT04619680	[36]	IV	120	Multicenter, randomized, placebo-controlled 150 mg POBID for 180 days
Pirfenidone	NCT04282902	[37]	III	294	Single-center, randomized, placebo-controlled 2 × 267 mg POTID for 4 weeks
	NCT04607928	[38]	II	148	Multicenter, randomized, placebo-controlled 2 × 267 mg POTID, 7 days after 4 × 267 mg TID for 24 weeks
Treamid	NCT04527354	[39]	II	60	Multicenter, randomized, placebo-controlled study 50 mg daily PO for 4 weeks
LYT-100	NCT04652518	[40]	II	168	Multicenter, randomized, placebo-controlled PO BID for 91 days
Collagen-Polyvinylpyrrolidone	NCT04517162	[41]	I	90	Single-center, randomized, placebo-controlled 1.5 mL IM BID for 3 days, then 1.5 mL QD for 4 days
Prednisone	NCT04551781	[42]	–	450	Single-center, randomized, placebo-controlled 20 mg daily for 14 IM
Bovhyaluronidase azoximer	NCT04645368	[43]	–	160	Multicenter, randomized, placebo-controlled 3000 ME IM once in 5 days for 15 IM
BIO 300 (genistein)	NCT04482595	[44]	II	66	Single-center, randomized, placebo-controlled 1500 mg daily PO for 12 weeks
Tetrandrine	NCT04308317	[45]	IV	60	Single-center, randomized, compared to standard therapy 60 mg daily PO for a week
Fuzheng Huayu Tablet	NCT04279197	[46]	II	160	Single-center, randomized, placebo-controlled 1.6 g TID PO for 24 weeks
Anluohuaxian	NCT04334265	[47]	–	750	Multicenter, randomized, compared to standard therapy 6 g BIDPO for 3 months
Stromal Vascular Fraction	NCT04326036	[48]	I	10	Single-center, randomized, placebo-controlled IV for 6 months, No data for injection frequency
IN01Vaccine	NCT04537130	[49]	Ib	40	On first stage, IN01 is injected on days 1, 14, 28, 42, and 56, On support stage, vaccination is carried out every 2 months with the same dosage and regimen as during introduction, compared to the patients receiving standard therapy

## Data Availability

Not applicable.

## References

[1] Bazdyrev E.D. (2020). Coronavirus disease: A global problem of the 21st century. Complex Issues Cardiovasc. Dis..

[2] Lechowicz K., Drożdżal S., Machaj F., Rosik J., Szostak B., Zegan-Barańska M., Biernawska J., Dabrowski W., Rotter I., Kotfis K. (2020). COVID-19: The potential treatment of pulmonary fibrosis associated with SARS-CoV-2 infection. JCM.

[3] Li X., Shen C., Wang L., Majumder S., Zhang D., Deen M.J., Li Y., Qing L., Zhang Y., Chen C. (2021). Pulmonary fibrosis and its related factors in discharged patients with new corona virus pneumonia: A cohort study. Respir. Res..

[4] Ali R.M.M., Ghonimy M.B.I. (2021). Post-COVID-19 pneumonia lung fibrosis: A worrisome sequelae in surviving patients. Egypt. J. Radiol. Nucl. Med..

[5] Wu X., Liu X., Zhou Y., Yu H., Li R., Zhan Q., Ni F., Fang S., Lu Y., Ding X. (2021). 3-month, 6-month, 9-month, and 12-month respiratory outcomes in patients following COVID-19-related hospitalisation: A prospective study. Lancet Respir. Med..

[6] Wells A.U., Devaraj A., Desai S.R. (2021). Interstitial lung disease after COVID-19 infection: A catalog of uncertainties. Radiology.

[7] Vadász I., Husain-Syed F., Dorfmüller P., Roller F.C., Tello K., Hecker M., Morty R.E., Gattenlöhner S., Walmrath H.-D., Grimminger F. (2021). Severe organising pneumonia following COVID-19. Thorax.

[8] Cottin V., Lafitte C., Sénéchal A., Traclet J. (2021). Interstitial lung disease after COVID-19. Am. J. Respir. Crit. Care Med..

[9] Udwadia Z.F., Pokhariyal P.K., Tripathi A.K.R., Kohli A. (2021). Fibrotic interstitial lung disease occurring as sequelae of COVID-19 pneumonia despite concomitant steroids. Lung India.

[10] Rai D., Kumar S., Sahay N. (2021). Post-COVID-19 pulmonary fibrosis: A case series and review of literature. J. Fam. Med. Prim. Care.

[11] Tale S., Ghosh S., Meitei S.P., Kolli M., Garbhapu A.K., Pudi S. (2020). Post COVID-19 pneumonia pulmonary fibrosis. QJM.

[12] Lei P., Fan B., Mao J., Wei J., Wang P. (2020). The progression of computed tomographic (CT) images in patients with coronavirus disease (COVID-19) pneumonia: Running title: The CT progression of COVID-19 pneumonia. J. Infect..

[13] Fu Z., Tang N., Chen Y., Ma L., Wei Y., Lu Y., Ye K., Liu H., Tang F., Huang G. (2020). CT features of COVID-19 patients with two consecutive negative RT-PCR tests after treatment. Sci. Rep..

[14] Francone M., Iafrate F., Masci G.M., Coco S., Cilia F., Manganaro L., Panebianco V., Andreoli C., Colaiacomo M.C., Zingaropoli M.A. (2020). Chest CT score in COVID-19 patients: Correlation with disease severity and short-term prognosis. Eur. Radiol..

[15] Vasarmidi E., Tsitoura E., Spandidos D.A., Tzanakis N., Antoniou K.M. (2020). Pulmonary fibrosis in the aftermath of the COVID-19 era (Review). Exp. Med..

[16] Rai D.K., Sharma P., Kumar R. (2021). Post covid 19 pulmonary fibrosis. Is it real threat?. Indian J. Tuberc..

[17] Combet M., Pavot A., Savale L., Humbert M., Monnet X. (2020). Rapid onset honeycombing fibrosis in spontaneously breathing patient with COVID-19. Eur. Respir. J..

[18] Ahmad Alhiyari M., Ata F., Islam Alghizzawi M., Bint I Bilal A., Salih Abdulhadi A., Yousaf Z. (2021). Post COVID-19 fibrosis, an emerging complicationof SARS-CoV-2 infection. IDCases.

[19] Dadhwal R., Sharma M., Surani S. (2021). Restrictive lung disease in patients with subclinical coronavirus infection: Are we bracing ourselves for devastating sequelae?. Cureus.

[20] Udwadia Z.F., Koul P.A., Richeldi L. (2021). Post-COVID lung fibrosis: The tsunami that will follow the earthquake. Lung India.

[21] Chun H.J., Coutavas E., Pine A., Lee A.I., Yu V., Shallow M., Giovacchini C.X., Mathews A., Stephenson B., Que L.G. (2021). Immuno-fibrotic drivers of impaired lung function in post-acute sequelae of SARS-CoV-2 infection (PASC). medRxiv.

[22] Zhou M., Yin Z., Xu J., Wang S., Liao T., Wang K., Li Y., Yang F., Wang Z., Yang G. (2021). Inflammatory profiles and clinical features of COVID-19 survivors three months after discharge in Wuhan, China. J. Infect. Dis..

[23] Qin W., Chen S., Zhang Y., Dong F., Zhang Z., Hu B., Zhu Z., Li F., Wang X., Wang Y. (2021). Diffusion capacity abnormalities for carbon monoxide in patients with COVID-19 at 3-month follow-up. Eur. Respir. J..

[24] McDonald L.T. (2021). Healing after COVID-19: Are survivors at risk for pulmonary fibrosis?. Am. J. Physiol. Lung Cell Mol. Physiol..

[25] Hui D.S., Joynt G.M., Wong K.T., Gomersall C.D., Li T.S., Antonio G., Ko F.W., Chan M.C., Chan D.P., Tong M.W. (2005). Impact of severe acute respiratory syndrome (SARS) on pulmonary function, functional capacity and quality of life in a cohort of survivors. Thorax.

[26] Hui D.S., Wong K.T., Ko F.W., Tam L.S., Chan D.P., Woo J., Sung J.J.Y. (2005). The 1-year impact of severe acute respiratory syndrome on pulmonary function, exercise capacity, and quality of life in a cohort of survivors. Chest.

[27] Wong K., Antonio G.E., Hui D.S.C., Ho C., Chan P., Ng W., Shing K., Wu A., Lee N., Yap F. (2004). Severe acute respiratory syndrome: Thin-section computed tomography features, temporal changes, and clinicoradiologic correlation during the convalescent period. J. Comput. Assist. Tomogr..

[28] Zhang P., Li J., Liu H., Han N., Ju J., Kou Y., Chen L., Jiang M., Pan F., Zheng Y. (2020). Correction: Long-term bone and lung consequences associated with hospital-acquired severe acute respiratory syndrome: A 15-year follow-up from a prospective cohort study. Bone Res..

[29] Ngai J.C., Ko F.W., Ng S.S., To K., Tong M., Hui D.S. (2010). The Long-term impact of severe acute respiratory syndrome on pulmonary function, exercise capacity and health status. Respirology.

[30] Park W.B., Jun K.I., Kim G., Choi J.-P., Rhee J.-Y., Cheon S., Lee C.H., Park J.-S., Kim Y., Joh J.-S. (2018). Correlation between pneumonia severity and pulmonary complications in middle east respiratory syndrome. J. Korean Med. Sci..

[31] Rivera-Ortega P., Hayton C., Blaikley J., Leonard C., Chaudhuri N. (2018). Nintedanib in the management of idiopathic pulmonary fibrosis: Clinical trial evidence and real-world experience. Adv. Respir. Dis..

[32] George P.M., Wells A.U. (2017). Pirfenidone for the treatment of idiopathic pulmonary fibrosis. Expert Rev. Clin. Pharm..

[33] Home—ClinicalTrials.Gov. https://clinicaltrials.gov/.

[34] (2020). Efficacy and Safety of Nintedanib Ethanesulfonate Soft Capsule in the Treatment of Pulmonary Fibrosis in Patients with Moderate to Severe COVID-9(COVID 19): A Single-Center, Randomized, Placebo-Controlled Study.

[35] (2020). Nintedanib for the Treatment of SARS-Cov-2 Induced Pulmonary Fibrosis.

[36] (2020). Early Nintedanib Deployment in COVID-19 Interstitial Fibrosis.

[37] (2020). A Randomized, Open-Label Study to Evaluate the Efficacy and Safety of Pirfenidone in Patients with Severe and Critical Novel Coronavirus Infection.

[38] (2020). Phase-II Randomized Clinical Trial to Evaluate the Effect of Pirfenidone Compared to Placebo in Post-COVID19 Pulmonary Fibrosis.

[39] (2020). Multicenter, Randomized, Double-Blind, Placebo-Controlled Pilot Study of Treamid Efficacy and Safety in the Rehabilitation of Patients After COVID-19 Pneumonia.

[40] (2020). A Phase 2 Randomized, Double-Blind, Placebo-Controlled Trial and Open Label Extension to Evaluate the Safety and Efficacy of Deupirfenidone (LYT-100) in Post-Acute COVID-19 Respiratory Disease.

[41] (2020). Effect of Collagen-Polyvinylpyrrolidone for the Treatment of Hyperinflammation and the Pulmonary Fibrosis in COVID-19 Patients. Double Blind Placebo-Controlled Pilot Trial.

[42] (2020). Short Term Low Dose Corticosteroids for Management of Post COVID-19 Pulmonary Fibrosis.

[43] (2020). Multicenter, Open-Label Prospective Cohort Study of the Efficacy and Safety of the Inclusion of Longidaze in the Prevention and Treatment of Post-Inflammatory Pulmonary Fibrosis and Interstitial Lung Diseases Caused by COVID-19.

[44] (2020). A Phase 2 Study of BIO 300 Oral Suspension in Discharged COVID-19 Patients.

[45] (2020). Clinical Study of Tetrandrine Tablets Adjuvant Treatment with COVID-19.

[46] Jing F., Fan H., Zhao Z., Xing F., He Y., Liu C. (2021). The efficacy of treating pulmonary fibrosis and pulmonary function injury in COVID-19 with the fuzheng huayu tablets: A multicenter randomized controlled trial. J. Dev. Drugs.

[47] Zhang C., Li J., Wu Z., Wang H., Que C., Zhao H., Wang G. (2020). Efficacy and safety of anluohuaxian in the treatment of rehabilitation patients with corona virus disease 2019-A multicenter, open, randomized controlled study. Trials.

[48] (2020). Use of CSVF for Residual Lung Damage (COPD/Fibrotic Lung Disease After Symptomatic COVID-19 Infection for Residual Pulmonary Injury or Post-Adult Respiratory Distress Syndrome Following Viral (SARS-Co-2) Infection.

[49] (2020). Phase Ib Controlled Exploratory Trial for Treatment of Fibrosing Interstitial Lung Disease Patients Secondary to SARS-CoV-2 Infection with IN01 Vaccine (COVINVAC).

[50] George P.M., Wells A.U., Jenkins R.G. (2020). Pulmonary fibrosis and COVID-19: The potential role for antifibrotic therapy. Lancet Respir. Med..

[51] Wollin L., Wex E., Pautsch A., Schnapp G., Hostettler K.E., Stowasser S., Kolb M. (2015). Mode of Action of Nintedanib in the treatment of idiopathic pulmonary fibrosis. Eur. Respir. J..

[52] Raghu G., Richeldi L. (2017). Current approaches to the management of idiopathic pulmonary fibrosis. Respir. Med..

[53] Yue X., Shan B., Lasky J.A. (2010). TGF-β: Titan of lung fibrogenesis. Curr. Enzyme Inhib..

[54] Margaritopoulos G.A., Vasarmidi E., Antoniou K.M. (2016). Pirfenidone in the treatment of idiopathic pulmonary fibrosis: An evidence-based review of its place in therapy. Core Evid..

[55] Wong A.W., Fidler L., Marcoux V., Johannson K.A., Assayag D., Fisher J.H., Hambly N., Kolb M., Morisset J., Shapera S. (2020). Practical considerations for the diagnosis and treatment of fibrotic interstitial lung disease during the coronavirus disease 2019 pandemic. Chest.

[56] Moore B.B., Moore T.A. (2015). Viruses in idiopathic pulmonary fibrosis. Etiology and exacerbation. Ann. Am. Thorac. Soc..

[57] Wootton S.C., Kim D.S., Kondoh Y., Chen E., Lee J.S., Song J.W., Huh J.W., Taniguchi H., Chiu C., Boushey H. (2011). Viral infection in acute exacerbation of idiopathic pulmonary fibrosis. Am. J. Respir. Crit. Care Med..

[58] (2020). Treatment with Pirfenidone for COVID-19 Related Severe ARDS an Open Label Pilot Trial.

[59] Tang N., Bai H., Chen X., Gong J., Li D., Sun Z. (2020). Anticoagulant treatment is associated with decreased mortality in severe coronavirus disease 2019 patients with coagulopathy. J. Thromb. Haemost..

[60] Guan W., Ni Z., Hu Y., Liang W., Ou C., He J., Liu L., Shan H., Lei C., Hui D.S.C. (2020). Clinical characteristics of coronavirus disease 2019 in China. N. Engl. J. Med..

[61] Skurikhin E., Nebolsin V., Widera D., Ermakova N., Pershina O., Pakhomova A., Krupin V., Pan E., Zhukova M., Novikov F. (2020). Antifibrotic and regenerative effects of treamid in pulmonary fibrosis. Int. J. Mol. Sci..

[62] National Center for Biotechnology Information PubChem Patent Summary for US-9504677-B2, Substituted N-aryl pyridinones. https://pubchem.ncbi.nlm.nih.gov/patent/US-9504677-B2.

[63] Olmos-Zuñiga J.R., Silva-Martínez M., Jasso-Victoria R., Baltazares-Lipp M., Hernández-Jiménez C., Buendía-Roldan I., Jasso-Arenas J., Martínez-Salas A., Calyeca-Gómez J., Guzmán-Cedillo A.E. (2017). Effects of pirfenidone and collagen-polyvinylpyrrolidone on macroscopic and microscopic changes, TGF-Β1 expression, and collagen deposition in an experimental model of tracheal wound healing. Biomed. Res. Int..

[64] Furuzawa-Carballeda J., Krötzsch E., Barile-Fabris L., Alcalá M., Espinosa-Morales R. (2005). Subcutaneous administration of collagen-polyvinylpyrrolidone down regulates IL-1beta, TNF-Alpha, TGF-Beta1, ELAM-1 and VCAM-1 expression in scleroderma skin lesions. Clin. Exp. Derm..

[65] Wilkinson E. (2020). RECOVERY Trial: The UK covid-19 study resetting expectations for clinical trials. BMJ.

[66] Yu W., Guo F., Song X. (2017). Effects and Mechanisms of pirfenidone, prednisone and acetylcysteine on pulmonary fibrosis in rat idiopathic pulmonary fibrosis models. Pharm. Biol..

[67] Lam E., Sayedy N., Anjum F., Akella J., Iqbal J. (2021). Corticosteroid therapy in post-COVID-19 pulmonary fibrosis. TP47. TP047 COVID and ARDS Case Reports.

[68] Myall K.J., Mukherjee B., Castanheira A.M., Lam J.L., Benedetti G., Mak S.M., Preston R., Thillai M., Dewar A., Molyneaux P.L. (2021). Persistent post–COVID-19 interstitial lung disease. An observational study of corticosteroid treatment. Ann. ATS.

[69] Novikova L.N., Zakharova A.S., Dzadzua D.V., Baranova O.P., Korzina N.V., Speranskaya A.A., Gichkin A.Y., Kameneva M.Y., Sukhovskaya O.A. (2011). Effects of longidaza in patients with idiopathic pulmonary fibrosis. Doctor.ru.

[70] BIO 300: A Promising Radiation Countermeasure under Advanced Development for Acute Radiation Syndrome and the Delayed Effects of Acute Radiation Exposure—PubMed. https://pubmed.ncbi.nlm.nih.gov/32450051/.

[71] Jackson I.L., Zodda A., Gurung G., Pavlovic R., Kaytor M.D., Kuskowski M.A., Vujaskovic Z. (2017). BIO 300, a nanosuspension of genistein, mitigates pneumonitis/fibrosis following high-dose radiation exposure in the C57L/J murine model. Br. J. Pharm..

[72] Para A.E., Bezjak A., Yeung I.W.T., Dyk J.V., Hill R.P. (2009). Effects of genistein following fractionated lung irradiation in mice. Radiother. Oncol..

[73] Ippolito E., Fiore M., Greco C., D’Angelillo R.M., Ramella S. (2020). COVID-19 and radiation induced pneumonitis: Overlapping clinical features of different diseases. Radiother. Oncol..

[74] Rios C.I., Cassatt D.R., Hollingsworth B.A., Satyamitra M.M., Tadesse Y.S., Taliaferro L.P., Winters T.A., DiCarlo A.L. (2021). Commonalities between COVID-19 and radiation injury. Radiat. Res..

[75] Costela-Ruiz V.J., Illescas-Montes R., Puerta-Puerta J.M., Ruiz C., Melguizo-Rodríguez L. (2020). SARS-CoV-2 infection: The role of cytokines in COVID-19 disease. Cytokine Growth Factor Rev..

[76] Narasaraju T., Tang B.M., Herrmann M., Muller S., Chow V.T.K., Radic M. (2020). Neutrophilia and NETopathy as key pathologic drivers of progressive lung impairment in patients with COVID-19. Front. Pharm..

[77] Hanania A.N., Mainwaring W., Ghebre Y.T., Hanania N.A., Ludwig M. (2019). Radiation-induced lung injury: Assessment and management. Chest.

[78] Gong L., Li Y., Nedeljkovic-Kurepa A., Sarkar F.H. (2003). Inactivation of NF-KappaB by genistein is mediated via akt signaling pathway in breast cancer cells. Oncogene.

[79] Liu T., Liu X., Li W. (2016). Tetrandrine, a Chinese plant-derived alkaloid, is a potential candidate for cancer chemotherapy. Oncotarget.

[80] Bhagya N., Chandrashekar K.R. (2016). Tetrandrine—A molecule of wide bioactivity. Phytochemistry.

[81] Kim D.E., Min J.S., Jang M.S., Lee J.Y., Shin Y.S., Park C.M., Song J.H., Kim H.R., Kim S., Jin Y.-H. (2019). Natural bis-benzylisoquinoline alkaloids-tetrandrine, fangchinoline, and cepharanthine, inhibit human coronavirus OC43 infection of MRC-5 human lung cells. Biomolecules.

[82] Dong H., Liu Y., Zhang J., Zhong W., Chen W., Cai S. (2020). Tetrandrine attenuates pulmonary fibrosis through Rheb/mTOR/p70S6k signaling mediated activation of autophagy. B64. Mechanistic Advances in Lung Fibrosis.

[83] Su W., Liang Y., Meng Z., Chen X., Lu M., Han X., Deng X., Zhang Q., Zhu H., Fu T. (2020). Inhalation of tetrandrine-hydroxypropyl-β-cyclodextrin inclusion complexes for pulmonary fibrosis treatment. Mol. Pharm..

[84] Borghardt J.M., Kloft C., Sharma A. (2018). Inhaled therapy in respiratory disease: The complex interplay of pulmonary kinetic Processes. Can. Respir. J..

[85] Liu W., Li Z., Sun Z., Xu Y., Wang S., Hu Y., Peng J. (2019). The components data of Fuzheng Huayu extracts, cordyceps sinensis mycelia polysaccharide, gypenosides and amygdalin. Data Brief.

[86] Dong S., Chen Q.-L., Su S.-B. (2015). Curative effects of Fuzheng Huayu on liver fibrosis and cirrhosis: A meta-analysis. Evid. Based Complementary Altern. Med..

[87] Tomaru A., Gabazza E., Kobayashi T., Kobayashi H., Taguchi O., Takagi T., Oonishi M., Fujiwara K., Gabazza C.D., Takahashi Y. (2015). Matrix metalloproteinase-2 is protective in bleomycin-induced pulmonary fibrosis. Eur. Respir. J..

[88] Tan S.-Z., Liu C.-H., Zhang W., Lu X., Ye W.-C., Cai Z.-Z., Liu P. (2007). Effects of Fuzheng Huayu recipe on MMP-2 activity and type IV collagen expression at fibrotic lung. Zhongguo Zhong Yao Za Zhi.

[89] Wu R., Dong S., Cai F.-F., Chen X.-L., Yang M.-D., Liu P., Su S.-B. (2019). Active compounds derived from Fuzheng Huayu formula protect hepatic parenchymal cells from apoptosis based on network pharmacology and transcriptomic analysis. Molecules.

[90] Ma W., Huang Q., Xiong G., Deng L., He Y. (2020). The protective effect of hederagenin on pulmonary fibrosis by regulating the Ras/JNK/NFAT4 axis in rats. Biosci. Biotechnol. Biochem..

[91] Walker N.M., Mazzoni S.M., Vittal R., Fingar D.C., Lama V.N. (2018). C-Jun N-terminal kinase (JNK)-mediated induction of MSin1 expression and MTORC2 activation in mesenchymal cells during fibrosis. J. Biol. Chem..

[92] Huang J., Huang H., Jiao Y., Ai G., Huang T., Li L., Yu H., Ma K., Xiao F. (2009). Effect of Anluohuaxian tablet combined with gamma-IFN on schistosomal liver fibrosis. J. Huazhong Univ. Sci. Technol. Med. Sci..

[93] Makarev E., Izumchenko E., Aihara F., Wysocki P.T., Zhu Q., Buzdin A., Sidransky D., Zhavoronkov A., Atala A. (2016). Common pathway signature in lung and liver fibrosis. Cell Cycle.

[94] Alexander R.W. (2020). Overview of COVID-19 lung damage clinical trial using Cellular Stromal Vascular Fraction (CSVF) and Functional Respiratory Imaging (FRI) analysis of pulmonary injury & post-viral (SARS=Cov-2) adult respiratory distress syndrome (ARDS). Ann. Stem Cell Res. Ther..

[95] Alexander R.W. (2020). Potential use of cellular stromal vascular fraction in Post-COVID-19 pulmonary injury and adult respiratory distress syndrome. J. Curr. Med. Res. Opin..

[96] Ntolios P., Manoloudi E., Tzouvelekis A., Bouros E., Steiropoulos P., Anevlavis S., Bouros D., Froudarakis M.E. (2018). Longitudinal outcomes of patients enrolled in a phase Ib Clinical trial of the adipose-derived stromal cells-stromal vascular fraction in idiopathic pulmonary fibrosis. Clin. Respir. J..

[97] Michalek J., Dudasova Z., Brown C. (2019). Stromal vascular fraction cell therapy for idiopathic pulmonary fibrosis—Cure without side effects. Ann. Clin. Case Rep..

[98] Tzouvelekis A., Ntolios P., Karameris A., Vilaras G., Boglou P., Koulelidis A., Archontogeorgis K., Kaltsas K., Zacharis G., Sarikloglou E. (2013). Increased expression of epidermal growth factor receptor (EGF-R) in patients with different forms of lung fibrosis. Biomed. Res. Int..

[99] Venkataraman T., Frieman M.B. (2017). The role of epidermal growth factor receptor (EGFR) signaling in SARS coronavirus-induced pulmonary fibrosis. Antivir. Res..

[100] Kućma M. (2020). Drug Candidate OATD-01 May Find Use in Treatment of Pulmonary Fibrosis in Patients Who Have Survived a New Coronavirus Infection (COVID-19). OncoArendi Ther. https://oncoarendi.com/en/oncoarendi-therapeutics-has-received-the-final-report-from-the-phase-ib-clinical-trial-of-innovative-drug-candidate-oatd-01/.

[101] Lee C.-M., He C.-H., Park J.W., Lee J.H., Kamle S., Ma B., Akosman B., Cotez R., Chen E., Zhou Y. (2019). Chitinase 1 regulates pulmonary fibrosis by modulating TGF-β/SMAD7 pathway via TGFBRAP1 and FOXO3. Life Sci Alliance.

[102] Dymek B., Sklepkiewicz P., Mlacki M., Zagozdzon A., Koralewski R., Mazur M., Paplinska-Goryca M., Nejman-Gryz P., Proboszcz M., Gorska K. (2018). CHIT1 Is a novel therapeutic target in Idiopathic Pulmonary Fibrosis (IPF): Anti-fibrotic efficacy of OATD-01, a potent and selective chitinase inhibitor in the mouse model of pulmonary fibrosis. Eur. Respir. J..

